# Trio-R: a script for assessing maternity and paternity in trio studies performed on Agilent chromosomal microarrays

**DOI:** 10.1186/s12911-018-0684-9

**Published:** 2018-11-06

**Authors:** Daniel Xia, Chen Zhang, Va Lip, Marian Harris, Yiping Shen

**Affiliations:** 1 Department of Pathology, University Health Network, 200 Elizabeth Street, Eaton Wing, 11th floor, Toronto, ON M5G 2C4 Canada; 2 Department of Pathology, Brigham and Women’s Hospital, Boston, MA USA; 3 Department of Pathology, Massachusetts General Hospital, Boston, MA USA; 4 Department of Laboratory Medicine, Boston Children’s Hospital, Boston, MA USA; 5 Claritas Genomics, Cambridge, MA USA; 6 Department of Pathology, Boston Children’s Hospital, Boston, MA USA

**Keywords:** Chromosomal microarrays, Trio studies, Single nucleotide polymorphisms, Non-paternity, Pre-analytical errors

## Abstract

**Background:**

Trio studies, which involve the testing of samples from a proband and both parents, are often used by clinical laboratories to help with the classification of genetic variants, including copy number variants. In order for the results of the trio study to be valid, the mother and father must be the true biological parents of the proband. As such, non-paternity and sample mix-ups are potential sources of error. To address these potential issues, we developed a computer script to accurately assess maternity and paternity using single nucleotide polymorphism (SNP) data generated by Agilent chromosomal microarrays, a platform-of-choice for clinical copy number testing.

**Results:**

We assessed the performance of the script on 10 putative trios tested at our laboratory, and found that the numbers and proportions of discordant SNPs were useful for determining parental relationships. The results of the assessment also confirmed maternity and paternity in the 10 trios tested, and by doing so essentially excluded pre-analytical sample switching in these 30 samples.

**Conclusions:**

Computational analysis of SNP data can be implemented as a quality control measure for trio testing performed on Agilent microarrays.

**Electronic supplementary material:**

The online version of this article (10.1186/s12911-018-0684-9) contains supplementary material, which is available to authorized users.

## Background

Array comparative genomic hybridization (aCGH) is a first-line diagnostic tool for the post-natal evaluation of patients with intellectual disability, autism spectrum disorders, and complex syndromes with multiple congenital anomalies [1]. A significant subset of copy number variants (CNVs) detected via this type of testing may be classified initially as variants of uncertain significance (VUSs) due to limitations in the current state of medical knowledge. In order to further characterize these VUSs, our laboratory (Claritas Genomics, Cambridge, MA) and many others will perform additional testing on the parents of the proband (i.e. the patient, often infants or children), to determine if the variants in the proband are inherited or de novo. The rationale is that a de novo copy number variant seen only in the affected proband is more likely to be pathogenic, since the unaffected parents do not have the variant. When performing this type of trio testing, maternity and paternity are often assumed, but this is potentially problematic because the rates of non-paternity in the general population may not be negligible [2, 3]. Further, human errors resulting in sample switching can also occur. To address this, we describe in this short report the development and testing of a computer script that analyzes single nucleotide polymorphism (SNP) data generated by Agilent chromosomal microarrays; this SNP data is intended for the assessment of absence-of-heterozygosity in the setting of suspected or known consanguinity; our script re-purposes the data for the assessment of maternity and paternity.

## Implementation

The scripts were written for R (version 3.2.2), a free statistical computing software. In order to develop and test our algorithm, we identified 10 putative trios (each consisting of a proband, a mother, and a father, presumably correctly matched) who were tested in our laboratory over a period of several months on the Agilent Human Genome CGH + SNP 4x180K Microarrays (Santa Clara, CA, USA). Briefly, the testing platform determines (in addition to copy number data) the genotypes (e.g., AA, AT, or TT) at approximately 30 thousand polymorphic SNP loci across the genome, and records the data as a text table file.

The scripts assess the relationships between a proband and the putative parents in the following manner: 1) data files from a proband and one of his/her putative parents (either the mother or father) are merged; 2) SNP loci are then filtered to only include those that are homozygous in both the proband and the parent (i.e., all heterozygous loci and those associated with copy number gains or losses [e.g., A or AAA] in either the proband or the parent are removed); 3) for each remaining (2-copy) homozygous locus, the scripts determines if the genotypes of the proband and parent are identical. If these are identical (e.g. parent is AA; proband is AA), the SNP locus is “concordant.” If these are not identical, the SNP locus is “discordant” (e.g. parent is TT; proband is AA; discordant because a TT parent cannot have an AA child). The premise is that correct proband-parent pairings (henceforth, true-pairs) should have very few discordant SNP loci (theoretically zero; in actuality probably a small number, presumably due to noise in the assay), while incorrect proband-parent pairings (henceforth, false-pairs; e.g. cases of non-paternity or sample mix-up) should have significantly greater numbers of discordant loci, since SNPs included are polymorphic. Since the numbers of SNP loci remaining after filtering (homozygous in both the proband and the putative parent) may vary depending on a number of factors (e.g. may be higher when there is a higher degree of relatedness), we considered the proportions of discordant SNPs (rather the absolute numbers of discordant SNPs) to be the most reliable values for assessing parental relationships.

Two versions of the script were developed, a test script for assessing performance on putative trios (as described above) and a clinical script for potential clinical use. In the clinical script, a true-pair is called only if the proportion of discordant SNP loci is < 0.015 (a cut-off selected by one author [DX]; see Fig. 2 and Discussions); otherwise, a false-pair is called. A “true” trio is called only if both the putative mother-proband and father-proband pairs are determined to be true-pairs; otherwise, a “false” trio is called. The results from the script’s analysis are reported in a text table file.

## Results

**Fig. 1:**
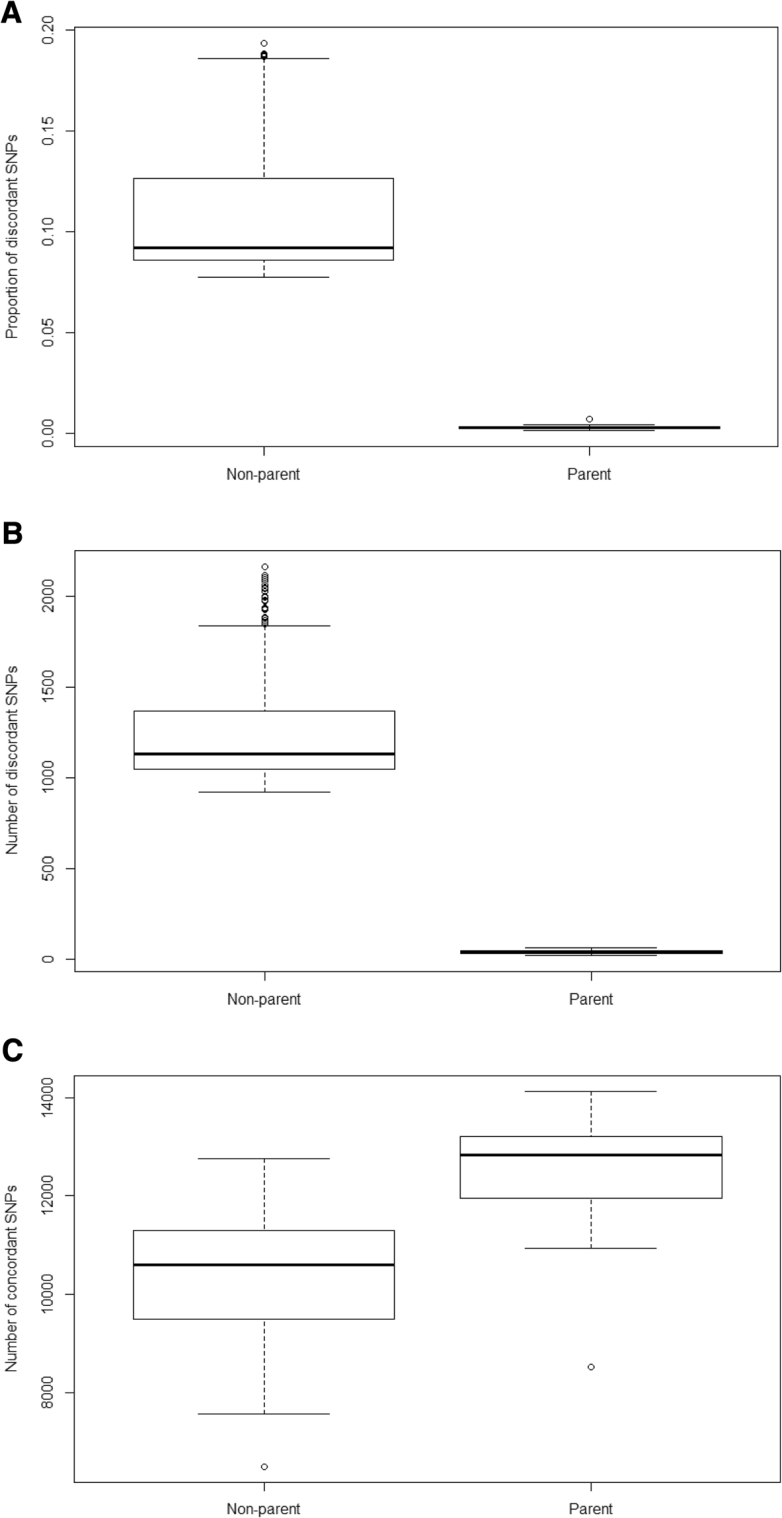
Performance of the script in a test involving 10 putative trios. **a** The distributions for the proportions of discordant loci for true-pairs (“Parents”; median = 0.0031, range = 0.0013–0.0072) and false-pairs (“Non-parents”; median = 0.092, range = 0.077 to 0.19) were clearly distinct. **b** A similar result was obtained for the numbers of discordant loci (for true-pairs/“Parents”; median = 39.5, range = 18–62; for false pairs/“Non-parents”; median = 1129, range = 920–2165). **c** By contrast, the numbers of concordant loci overlapped, suggesting that these do not reliably separate true-pairs from false-pairs. Overall, we considered the proportion of discordant loci to be the most reliable measure (see Implementation section)

We tested the performance of the script (test script) using real patient data from the 10 putative trios, and determined the numbers of 1) discordant SNP loci, 2) concordant SNP loci, and 3) proportion of discordant SNP loci across 20 possible putative true-pairs (10 matched mother-proband pairs, and 10 matched father-proband pairs) and 180 possible putative false-pairs (where each proband is artificially mismatched with all possible combinations of incorrect parents). This analysis (Fig. 1) demonstrated 1) that the number of discordant SNPs and the proportion of discordant SNPs can accurately separate true-pairs from false pairs, 2) that there were no cases of non-paternity in the 10 trios tested, and 3) that by confirming parental status, pre-analytical sample-switching in the 10 trios (30 samples) was largely excluded.

## Discussion

Confirmation of paternity and maternity is required to prove variant de novo status, which is in turn important for genetic variant classification. In guidelines from the American College of Medical Genetics and Genomics, de novo sequence variants with confirmed maternity and paternity are regarded as strong evidence in support of pathogenicity, while de novo variants with assumed maternity and paternity are regarded as moderate evidence [4]. Current guidelines for de novo copy number variants do not distinguish assumed from confirmed parental relationships in this manner [1], but a similar rationale applies [5].

Our clinical script performs quality control functions that overlap with the quality control modules of the Affymetrix GeneChip Genotyping (GTYPE) Analysis Software (http://www.affymetrix.com/support/technical/datasheets/gtype_datasheet.pdf) and SNPTrio [6]. To our knowledge, however, this script is the first computational tool available for assessing maternity and paternity on the Agilent microarray platform, a method-of-choice for copy number testing.

There are a number of potential issues related to the validation and use of the script. First, we did not include close relatives of the probands/parents as part of the validation. The proportion of discordant SNPs in any analysis depends on a number of factors including: 1) the degree-of-relatedness between the putative parent and the proband, 2) the frequencies of the alleles for each SNPs on the array in different populations, and 3) the quality metrics of the specimens, which may vary from laboratory to laboratory. In view of these considerations, we cannot guarantee that the script will reliably distinguish parents from close relatives in all conceivable laboratory settings. Also, the script may be unreliable in other situations. For example, the algorithm will fail to detect a sample switch between the proband and his/her brother or sister, since sibling-parent pairs are true-pairs. Overall, we would recommend that clinical laboratories consider independent internal validation studies (possibly involving samples from close relatives) prior to using script; further, any unusual results reported by the script (e.g., a value intermediate between the expected values for true-parents and unrelated strangers) should be treated with caution.

**Fig. 2:**
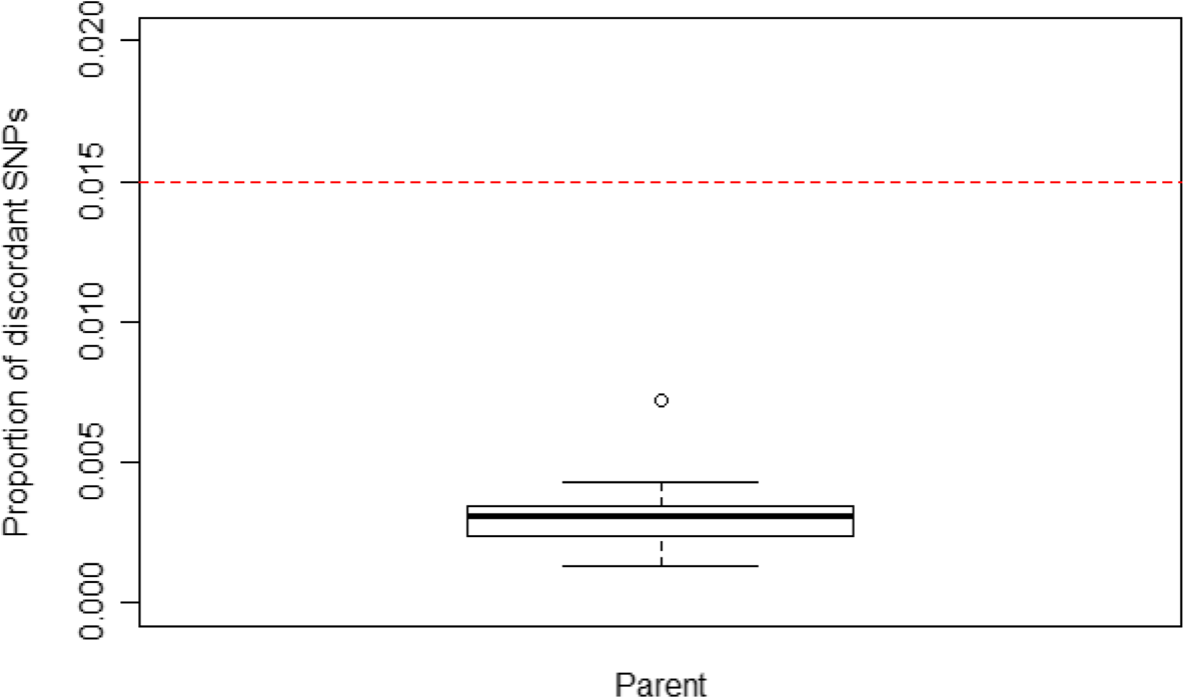
The selected cut-off for calling a true pair (proportion of discordant SNPs < 0.015, red-dashed line) in relation to the corresponding distribution for true pairs

A second potential issue is that some aspects of our relatively simple script may not be optimized. For example, the cut-off for calling true-pairs (proportion of discordant SNPs < 0.015) in this study is not the result of the statistical analyses of well-defined distributions. The value was selected based on the proportions of discordant SNPs observed for the 20 true proband-mother and proband-father pairs (Fig. 2). The cut-off is approximately 9.5 standard deviations above the mean for that distribution, but it is uncertain if the distributions are truly Gaussian or if the cut-off is optimal. The script also does not use identical-by-descent or segregated segment approaches available in more advanced computational tools, such as PLINK [7]. Despite these potential limitations, however, we expect the algorithm as described to perform well for the confirmation of parental status in trio studies performed on the Agilent platform, in the vast majority of clinical scenarios.

The clinical script (under GPLv3 license) and protocol/instructions are available as Additional files 1 and 2 and at our web site (see Availability and Requirements below). The user is required to install R (and optionally RStudio). Control data files from our laboratory are not available because these contain patient genetic data; however, interested laboratories should be able to easily create controls from real patient files. Although designed to process data from Agilent microarrays, the script could be potentially adapted for other platforms, and for other related tasks (e.g. to identify the true parents of any given proband from a list of candidate samples using SNP data, in the setting of a sample mix-up).

## Conclusions

Computational analysis of SNP data, originally intended for the assessment of absence of heterozygosity, can be implemented as a quality control measure for trio studies performed using Agilent microarrays.

## Availability and requirements

**Project name:** Trio-R: A Script for Assessing Maternity and Paternity in Trio Studies Performed on Agilent Chromosomal Microarrays.

**Project home page:** https://sites.google.com/site/trioscript/

**Operating system:** Platform independent.

**Programming language:** R.

**Other requirements:** R (developed using version 3.2.2).

**Any restrictions to use by non-academics:** none.

## Additional files

Additional file 1: Protocol for implementing the R-script. (DOCX 167 kb)

Additional file 2: Clinical version of the R-script. (R 7 kb)

## Abbreviations

aCGH: Array comparative genomic hybridization
CNV: Copy number variant
SNP: Single nucleotide polymorpishm
VUS: Variant of uncertain significance
