# Supplementary material for: Trio-R: a script for assessing maternity and paternity in trio studies performed on Agilent chromosomal microarrays
Source: BMC Med Inform Decis Mak. 2018 Nov 6;18:91. doi: 10.1186/s12911-018-0684-9 (PMC6220459; doi:10.1186/s12911-018-0684-9)
Supplement: Supplementary file 1 — Protocol for implementing the R-script. (DOCX 167 kb) [file 12911_2018_684_MOESM1_ESM.docx]

**Protocol for Trio-R: A R script for Assessing Maternity and Paternity Using SNP Data from Agilent Chromosomal Microarrays**

**GNU GPL v3 License:**

Trio-R: A R script for Assessing Maternity and Paternity Using SNP Data from Agilent Chromosomal Microarrays

Copyright (C) 2015 Daniel Xia, Cheng Zhang, Va Lip, Marian Harris, Yiping Shen

This program is free software: you can redistribute it and/or modify it under the terms of the GNU General Public License as published by the Free Software Foundation, either version 3 of the License, or (at your option) any later version.

This program is distributed in the hope that it will be useful, but WITHOUT ANY WARRANTY; without even the implied warranty of MERCHANTABILITY or FITNESS FOR A PARTICULAR PURPOSE. See the GNU General Public License for more details.

You should have received a copy of the GNU General Public License along with this program. If not, see <http://www.gnu.org/licenses/>.

**Introduction and purpose:** The assessment of *de novo* copy number variants (CNVs) in trio studies in a proband requires, under ideal circumstances, the confirmation of maternity and paternity in parental reference samples. Although relatively uncommon, pre-analytical errors (e.g. sample switching) and instances of non-paternity can happen, and these can adversely impact the interpretation of *de novo* germline variants in trio studies.

This script is intended as a quality control for array comparative genomic hybridization (aCGH) trio testing in clinical genetics laboratories. The script involves computational analyses of single nucleotide polymorphism (SNP) data generated by Agilent microarrays from probands and the putative parents.

The script is written for R (developed using version 3.2.2). Data for approximately 30 thousand SNPs from a proband and his/her putative parent (either mother or father) are combined. The SNPs are then filtered to include only those that are homozygous in both the proband and the parent (see also note below). For each included SNP, the script determines if the genotypes of the proband and parent are identical.

- If they are identical (e.g. parent is AA; proband is AA), the SNP is concordant.
- If not identical (e.g. parent is TT; proband is AA), the SNP is discordant.

The premise for the analysis is that true proband-parent pairs (true pairs) should have very few discordant SNPs, while false proband-parent pairs (false pairs) should have many more discordant SNPs.

A note about filtering homozygous SNPs:

By restricting the analysis to homozygous SNPs, assay “noise” is theoretically reduced. In particular, the platform should have less trouble distinguishing two different homozygous calls (e.g., AA from TT, i.e. high vs low signals), than homozygous from heterozygous calls (e.g., AA from AT, i.e. high or low vs intermediate signals; see image below, taken from the Genetic Engineering and Biotechnology web site <http://www.genengnews.com/gen-articles/improving-loh-detection-on-cgh-microarrays/3464?page=2>).


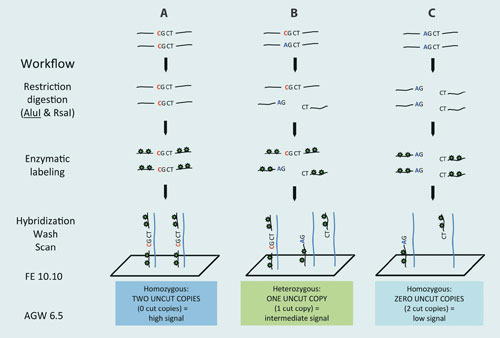


Please note that this filtering step also removes SNPs associated with copy number losses and gains (e.g., A or AAA), since only 2-copy homozygous calls in the proband and putative parent are retained.

**Computer software and data files:**

1. R download: <https://cran.rstudio.com/>
2. RStudio download (optional): <https://www.rstudio.com/products/RStudio/>
3. C:\Your Favorite Folder. This is the folder that contains the SNP data files to be analyzed, and where the script and protocol is stored.

**Quality control:** Reference SNP data files for a known true trio (a proband with confirmed true parents) and an artificially created false trio (a proband with artificially mismatched false parents) are suggested. Because the SNP files contain patient genetic data, reference files from our laboratory is not provided. The assumption is that reference files can be easily created from existing patient data files from any laboratory interested in implementing this script.

**Step 1: Installing R and RStudio**

***Note:*** *Installation of R and RStudio (Step 1) is only necessary the first time this protocol is performed on a computer.*

1. To install R, go to: <https://cran.rstudio.com/>
2. Click the link “Download R for Windows” (or Linux or MacOS, depending on the operating system).
3. Open the download folder and run the installation program.
4. Follow the instructions during installation. Install the program with default settings.
5. To install R-Studio, go to: <https://www.rstudio.com/products/RStudio/>
6. Click the link “RStudio Desktop”
7. On the next page, choose the “Open Source Edition”
8. Open the download folder and run the installation program.
9. Follow the instructions during installation. Install the program with default settings.

***Note:*** *RStudio is not required to run R scripts, but some may find that the software makes R easier to use. The remainder of the instructions assumes that you have RStudio installed. You may want to modify protocol if you run a different software.*

**Step 2: Renaming the SNP data files**

1. Go to the directory where the proband, mother, and father SNP files are saved.
2. Open the appropriate "XLS" file, and save it as a "TXT" in C:\Your Favorite Folder.

***Note:*** *For this version of the script, the saved file names must be in following format:
a. Proband files must have the format: proband*.txt (e.g. proband1.txt).
b. Mom files must have the format: mom*.txt (e.g. mom1.txt).
c. Dad files must have the format: dad*.txt (e.g. dad1.txt).
d. The ‘*’ portion of saved file names for each trio must match (e.g. proband1.txt, mom1.txt, and dad1.txt is one trio, where the “1” part of the file names match).*

3. Repeat 1-2 as needed for SNP files from all the proband(s), mom(s), and dad(s) to be analyzed.

**Step 3: Running the analysis**

1. Go to C:\Your Favorite Folder and open the “trio.confirmation.R” script.

***Note:*** *The first time the script is run on a computer, you may need to associated *.R files with R or RStudio.*

2. In RStudio, the script will open in the left upper workspace. To run the script, single click the left upper workspace. Press Ctrl+A to select all the code and press Ctrl+Enter to run the selected code.

***Note:*** *It is important to run the entire script from the beginning. Failing to do so could lead to errors in the analysis.*

3. The script will produce the results from the analysis in the left lower display. The same results will also be saved to a CSV file, “True trio analysis results.csv,” which is a text table file that you can open in Excel (See Figure).


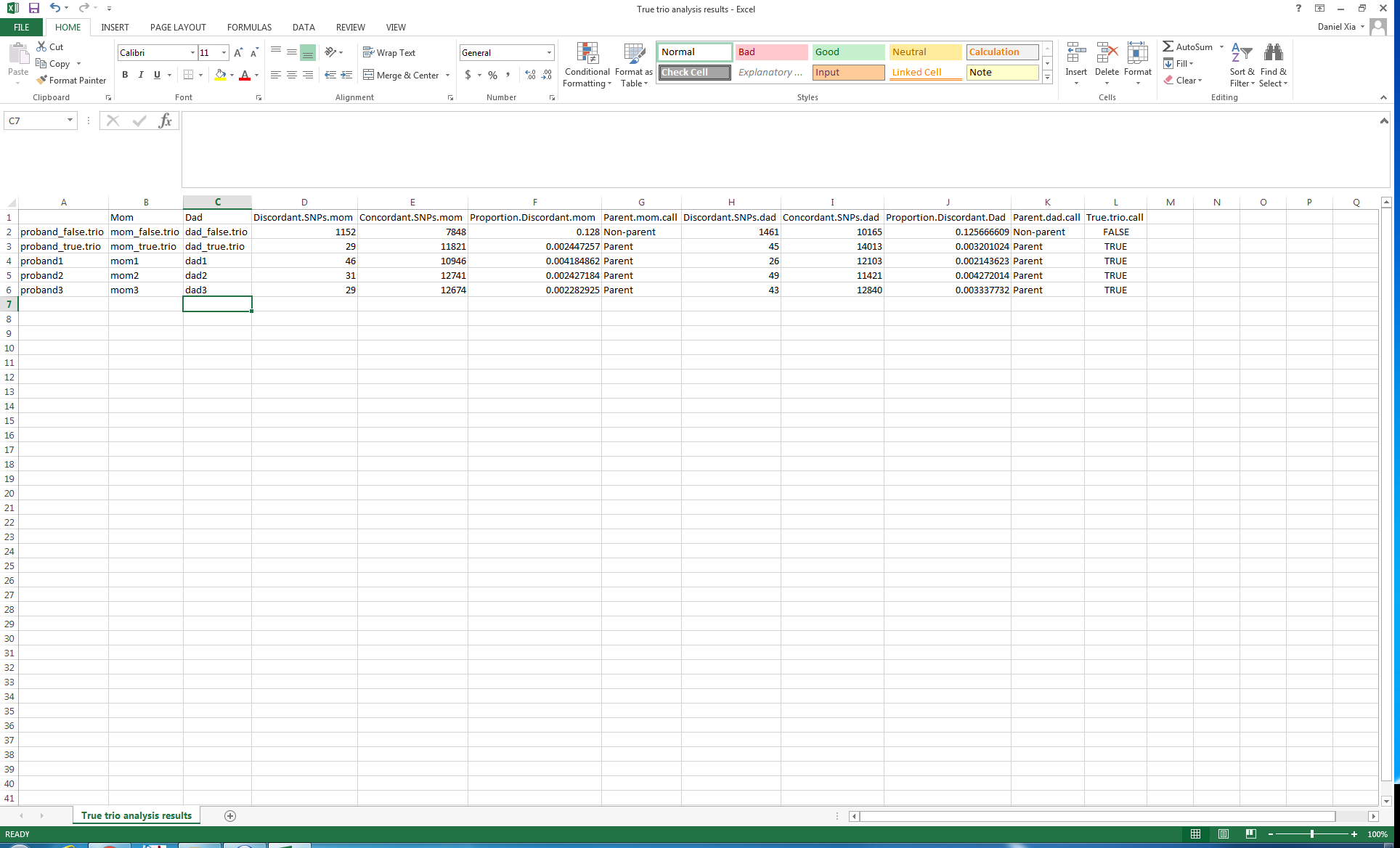


**Step 4: Technical review of results**

1. Close RStudio and open the “True trio analysis results.csv” file in Excel.

***Note:*** *The R-script uses the following criteria to distinguish parents from non-parents:
“Parent” = “Proportion discordant SNPs < 0.015”
Otherwise, “Non-parent”
These thresholds are selected by one author (DX) on the basis of internal validation. Other cutoffs may be more appropriate at other laboratories.

The R-script uses the following criteria to distinguish true trios from false trios:
“TRUE” = “Mom = Parent” AND “Dad = Parent”
Otherwise, “FALSE”*

2. Review the results for the trio(s) in question and the controls.
3. Optionally, one could consider, at the end of the analysis, copying all of the files from C:\Your Favorite Folder (including protocol and control data files) into an appropriately named subfolder with the date of the analysis (e.g. Trio Confirmation, 12.11.15) as a complete record of the analysis performed.

4. Delete the proband, mom, and dad SNP files for the trios saved in Step 2 (see above; items 1-2).

***Note:*** *Failing to delete the data file from the current analysis may lead to errors the next time the script is run. Do not delete the control SNP files or other files (e.g. protocol, R-script, etc.) in the folder.*
